# Metal-binding polymorphism in late embryogenesis abundant protein AtLEA4-5, an intrinsically disordered protein

**DOI:** 10.7717/peerj.4930

**Published:** 2018-06-07

**Authors:** Leidys French-Pacheco, Cesar L. Cuevas-Velazquez, Lina Rivillas-Acevedo, Alejandra A. Covarrubias, Carlos Amero

**Affiliations:** 1 Centro de Investigaciones Químicas, IICBA, Universidad Autónoma del Estado de Morelos, Cuernavaca, Morelos, Mexico; 2 Departamento de Biología Molecular de Plantas, Instituto de Biotecnología, Universidad Nacional Autónoma de México, Cuernavaca, Morelos, Mexico; 3 Centro de Investigación en Dinámica Celular, IICBA, Universidad Autónoma del Estado de Morelos, Cuernavaca, Morelos, Mexico

**Keywords:** Intrinsically disordered proteins, Metal binding, Protein self-assembly, Fuzzy complex

## Abstract

Late embryogenesis abundant (LEA) proteins accumulate in plants during adverse conditions and their main attributed function is to confer tolerance to stress. One of the deleterious effects of the adverse environment is the accumulation of metal ions to levels that generate reactive oxygen species, compromising the survival of cells. AtLEA4-5, a member of group 4 of LEAs in *Arabidopsis*, is an intrinsically disordered protein. It has been shown that their *N*-terminal region is able to undergo transitions to partially folded states and prevent the inactivation of enzymes. We have characterized metal ion binding to AtLEA4-5 by circular dichroism, electronic absorbance spectroscopy (UV–vis), electron paramagnetic resonance, dynamic light scattering, and isothermal titration calorimetry. The data shows that AtLEA4-5 contains a single binding site for Ni(II), while Zn(II) and Cu(II) have multiple binding sites and promote oligomerization. The Cu(II) interacts preferentially with histidine residues mostly located in the C-terminal region with moderate affinity and different coordination modes. These results and the lack of a stable secondary structure formation indicate that an ensemble of conformations remains accessible to the metal for binding, suggesting the formation of a fuzzy complex. Our results support the multifunctionality of LEA proteins and suggest that the C-terminal region of AtLEA4-5 could be responsible for antioxidant activity, scavenging metal ions under stress conditions while the *N*-terminal could function as a chaperone.

## Introduction

Late embryogenesis abundant (LEA) proteins are highly expressed and accumulated in plants during the dehydration stage of seeds and pollen, but also in response to water deficit conditions and to other adverse environments (Dure et al., 1983; Battaglia et al., 2008; Hand et al., 2011). These characteristics have suggested that their main role is to confer tolerance to stress conditions (Kovács, Agoston & Tompa, 2008; Hincha & Thalhammer, 2012). LEA proteins are grouped in seven families (LEA1–LEA7) according to their sequence similarity, phylogeny, and characteristic motifs (Battaglia et al., 2008). Most of them are classified as intrinsically disordered proteins (IDPs), due in part to their high content of small, polar, and charged amino acids (Garay-Arroyo et al., 2000; Battaglia et al., 2008; Olvera-Carrillo, Reyes & Covarrubias, 2011; Hincha & Thalhammer, 2012). Experimental evidence by circular dichroism (CD) and nuclear magnetic resonance have confirmed large amounts of disordered conformations for some members (Rivera-Najera et al., 2014; Cuevas-Velazquez et al., 2016). However, in the presence of α-helix inducers as trifluoroethanol LEA proteins may undergo transitions to partially folded states. This phenomenon also occurs under dehydration and conditions that simulate low osmotic potentials or macromolecular crowding (Soulages et al., 2002; Cuevas-Velazquez et al., 2016; Bremer et al., 2017).

To promote tolerance of plants, they may act as chaperone-like proteins by interacting with other proteins to preclude the inactivation and subsequent aggregation that they experience under stress conditions (Reyes et al., 2005; Chakrabortee et al., 2007; Kovács, Agoston & Tompa, 2008; Battaglia & Covarrubias, 2013). There is also evidence indicating that some LEA proteins may be involved in the stabilization of biological membranes (Koag et al., 2009; Bremer et al., 2017) and in nucleic acids protection by direct interaction (Hara et al., 2009; Saumonneau et al., 2012). Furthermore, it has been reported that LEA proteins from different families are able to bind metals, suggesting a role as metal ion sequestering agents (Krüger et al., 2002; Liu et al., 2011; Hara et al., 2016).

In plants, metal ions can accumulate to levels that generate reactive oxygen species (ROS), compromising the survival of cells (DalCorso et al., 2014). ROS might be produced by free catalytic metal as Cu(II), Zn(II) or Fe(III) via the Haber–Weiss and the Fenton reactions or metabolic alterations (Letelier et al., 2005; Ravet & Pilon, 2013; DalCorso et al., 2014). The putative interaction of LEA proteins with metal ions suggests that they might prevent the deleterious effects caused by the accumulation of such molecules (Hara, Fujinaga & Kuboi, 2004; Liu et al., 2011). Previous works have reported interaction with Zn(II), Ni(II), Cu(II), and Fe(III), for CuCOR15 a member of group 2 from citrus (Hara, Fujinaga & Kuboi, 2005); ZmLEA3 a member of group 3 from maize (Liu et al., 2013) and GmPM1 and GmPM9 members of group 4 from soybean (Liu et al., 2011). Interestingly, GmPM1 and GmPM9 bind Fe(III) and Cu(II) but not Ca(II) or Mg(II) ions, and hence it is not clear if the metal binding and specificity is conserved in each group or if it is protein-specific.

**Figure 1:**
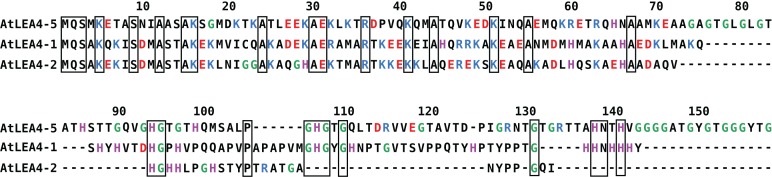
Sequence alignment for group 4 LEA proteins from *Arabidopsis thaliana*: AtLEA4-5, AtLEA4-1, and AtLEA4-2. According to the nomenclature of LEA proteins, numbers 1, 2, and 5 indicate the chromosome where their corresponding genes are localized. Fully conserved residues are contained in black boxes, positive charged residues are in blue, negative charged residues are in red, glycines are in green and histidines are in purple. Their amino terminal region is highly conserved, whereas their carboxyl terminal region is more variable, with 54.64% sequence identity between AtLEA4-1 and AtLEA4-2, 29.69% between AtLEA4-1 and AtLEA4-5, and 26.09% between AtLEA4-2 and AtLEA4-5.

In *Arabidopsis thaliana*, the group 4 of LEA proteins is formed by three basic and hydrophilic non-redundant proteins: AtLEA4-1, AtLEA4-2, and AtLEA4-5, that have been classified as IDPs (Olvera-Carrillo et al., 2010; Shih et al., 2010). Their amino terminal region is highly conserved, whereas their carboxyl terminal region is more variable, with 54.64% sequence identity between AtLEA4-1 and AtLEA4-2, 29.69% between AtLEA4-1 and AtLEA4-5 and 26.09% between AtLEA4-2 and AtLEA4-5 (Fig. 1). It has been shown that two members of this family, AtLEA4-5 and AtLE4-2, undergo transitions to partially folded states, mainly alpha helices as determined by CD, under conditions that mimic low osmotic potentials or high macromolecular crowding simulated by different concentrations of glycerol or polyethylene glycol, respectively (Cuevas-Velazquez et al., 2016). Also, both proteins prevent the inactivation and/or the aggregation of enzymes under partial dehydration or freeze-thaw treatments (Reyes et al., 2008; Cuevas-Velazquez et al., 2016). Previous analysis showed that besides the characteristic amino acid composition of typical LEA proteins, group 4 LEA proteins present a particular bias towards a high content of positively charged residues (Arg and Lys) in their amino terminal region, whereas their carboxy terminal region show a high percentage of Gly and His residues (Fig. 1) (Cuevas-Velazquez, Reyes & Covarrubias, 2017).

To gain insight into the properties of the Arabidopsis group 4 LEA proteins, we have characterized the metal binding by several biophysical techniques, such as CD, electronic absorbance spectroscopy (UV–vis), electron paramagnetic resonance (EPR), dynamic light scattering (DLS), and isothermal titration calorimetry (ITC). In this work, we show that AtLEA4-5 is able to bind Zn(II), Cu(II), and Ni(II) but not Ca(II), Mn(II), and Fe(III), and describe the metal binding properties for this protein.

## Materials and Methods

All reagents were analytic grade, and used without further purification. The water used was MQ grade. CuCl_2_ and CuSO_4_, MnCl_2_, FeCl_3_, CaCl_2_, NiSO_4_, and ZnSO_4_ were used as source of Cu(II), Mn(II), Fe(III), Ca(II), Ni(II), and Zn(II) ions, respectively.

### Protein expression and purification

The AtLEA4-5/pTrc99A plasmid was transformed into the *Escherichia coli* D3-lysS strain (Cuevas-Velazquez et al., 2016). Bacterial cells were grown in 1 L of LB, supplemented with 100 μg mL^−1^ ampicillin at 37 °C. The recombinant protein expression was induced by addition of 0.3 mM isopropyl-d-thiogalactopyranoside at OD_600_ = 0.6, and harvested by centrifugation after incubation during 6 h at 25 °C. The cell pellet was resuspended in 20 mM Tris–HCl, pH 7, 10 mM NaCl and lysed on ice by sonication. AtLEA4-5 was purified by thermic treatment of the bacterial protein extract, followed by differential precipitation with trichloroacetic acid, as previously described (Campos et al., 2011).

### Immobilized metal ion affinity chromatography

The interaction with metal ions was analyzed by immobilized metal ion affinity chromatography (IMAC) using 1 mL HiTrap chelating HP columns following manufacturer instructions (GE Healthcare Life Science, Princeton, NJ, USA).

The columns were charged with different metal ions by loading 5 mL of 100 mM of MnCl_2_, CaCl_2_, FeCl_3_, ZnSO, CuSO_4_ or NiSO_4_, and washed with 10 mL of deionized water. Every column was equilibrated with 50 mM Tris–HCl pH 7.5, 1M NaCl, except the one that contains FeCl_3_, which was equilibrated at 50 mM Tris–HCl pH 5.5. A total of 50 μM of protein was loaded into the columns and then washed with 8 mL of 50 mM Tris–HCl pH 7.5, 1M NaCl. The bound protein was eluted with 4 mL of 50 mM Tris–HCl pH 7.5, 1M NaCl 250 mM Ethylenediaminetetraacetate acid (EDTA). Every fraction was analyzed by Sodium dodecyl sulfate polyacrylamide (SDS–PAGE).

### UV–visible absorption

Electronic absorption spectra were acquired on an Agilent 8453 UV–visible diode array spectrophotometer at room temperature. AtLEA4-5 protein samples were 120 μM in either 10 mM PBS, pH 7.5 or 10 mM NEM (4-ethylmorpholine) pH 7.5. Titration steps of 0.5 equivalents of metal ions were used to analyze the formation of protein-metal complexes. Changes were observed between 200 and 800 nm. Duplicates experiments were performed for each condition. NEM buffer was chosen due to its non-chelating properties (Garnett & Viles, 2003).

### Circular dichroism

The formation of the metal-protein complex was monitored by CD in the presence of metal ions. Spectra were acquired on a Jasco J-815 CD spectropolarimeter, using a 1 cm path length quartz cuvette at room temperature. A 120 μM AtLEA4-5 sample in 10 mM NEM, pH 7.5 was titrated by the addition of 0.5 equivalents of CuSO_4_.

Secondary structure changes induced by the protein-metal ion interaction were followed by the addition of increasing amounts of metal ions. The spectra in the far UV region (200–250 nm) were recorded at each titration point. Duplicates experiments were performed for each condition.

### Electron paramagnetic resonance

Paramagnetic resonance spectra were collected in the X-band microwave frequency (9.5 GHz) using an EMX Plus Bruker System, at 150 K with a ER4131VT variable temperature nitrogen system. The samples were run using 10 mW microwave power, 5 G modulation amplitude, 100 kHz modulation frequency, 327 ms time constant, and 82 ms conversion time. AtLEA4-5 samples were 120 μM in 10 mM NEM, pH 7.5 and progressively increasing equivalents of CuSO_4_ metal ions were added. Duplicates experiments were performed for each condition.

### Absorption binding calculation

To get an estimation of the binding affinity, the average EPR signal from *g* = 2.12 and *g* = 2.45 were plotted as a function of Cu(II) concentration. The normalized values were analyzed by non-linear fitting using a hyperbolic equation:

$$y = {{p\left[ {{\rm{Cu}}} \right]} \over {\left({{k_{\rm{b}}} + \left[ {{\rm{Cu}}} \right]} \right)}}$$

where *y* is the absorbance, [Cu] is the copper concentration, *p* is the maximum specific binding, and *k*_b_ is the apparent binding constant.

### Isothermal titration calorimetry

Isothermal titration calorimetry experiments were performed at 25 °C on a Malvern ITC200 instrument. A total of 50 μM AtLEA4-5 in NEM 10 mM pH 7.5 was loaded into the sample cell and 1–4 mM of metal ion (CuCl_2_, ZnSO_4_ or NiSO_4_) solution was loaded into the syringe. Each experiment consisted of 20 injections of 2 μL of each metal ion with 180 s interval between injections and stirring at 750 rpm. The heat of dilution was determined by making identical injection in the absence of protein. The net reaction heat was obtained by subtracting the heat of dilution from the corresponding total heat of reaction. Triplicate experiments were performed for each condition with different parameters.

The thermograms were integrated with the NIPTIC software package (Keller et al., 2012). The baseline, the beginning and the end of each peak were adjusted automatically by the program. Then the data sets were fitted using a nonlinear least-squares algorithm to a binding model of either one or multiple sets of non-interacting binding sites by SEDPHAT (Zhao, Piszczek & Schuck, 2015). The binding enthalpy change Δ*H*, and the association constant Ka were permitted to float during the least-squares minimization process and taken as the best-fit values.

### Dynamic Light Scattering

Dynamic light scattering measurements were performed on a Malvern Zetasizer Nano ZSP spectrophotometer, with a scatter angle of 173°. AtLEA4-5 samples were 120 μM in 10 mM NEM, pH 7.5 in the absence and presence of 1, 2, 3 or 4 CuCl_2_ equivalents. Samples with 50 and 12 μM were also used. The data were used to obtain translational diffusion coefficients through measurement of the decay rates of scattered light (correlation function) (Stetefeld, McKenna & Patel, 2016). The hydrodynamic radius, *R*_H_, were obtained from the diffusion coefficients, *D*, via the Stokes–Einstein equation:

$${R_{\rm{H}}} = {{{K_{\rm{B}}}T} \over {6{\rm{\pi \eta }}D}}$$

where *K*_B_ is Boltzmann’s constant, *T* is the temperature, and η is the viscosity of the solution. We use 0.8872 cP for the viscosity at 25 °C. Typically, five runs with 10 scans of 10 s were obtained for each sample by triplicated. Data were analyzed by the cumulan and the distribution methods implemented in the SEDPHAT/SEDFIT software (Zhao, Piszczek & Schuck, 2015; Brautigam et al., 2016).

An estimated hydrodynamic radius, *R*_H_, for IDPs was calculated using (Tomasso et al., 2016):

$${R_{\rm{H}}} = {\rm{ }}\left({1.24{\rm{ }} \times {\rm{ }}{F_{{\rm{PRO}}}} + {\rm{ }}0.904} \right){\rm{ }} \times {\rm{ }}\left({0.00759{\rm{ }} \times {\rm{ }}\left| Q \right| + {\rm{ }}0.963} \right){\rm{ }} \times {\rm{ }}2.49{\rm{ }} \times {\rm{ }}{N^{0.509}}$$

where *N* is the residue number, *F*_PRO_ is the fractional number of proline residues, and |*Q*| is the absolute net charge determined from the sequence.

## Results

AtLEA4-5 was predicted to be disordered based on previous amino acid sequence analysis, while CD spectra showed a typical spectrum for a disorder protein, as indicated by the presence of minimum at ∼200 nm (Fig. S1), in agreement with previous reports (Cuevas-Velazquez et al., 2016). In order to determine qualitatively if AtLEA4-5 was able to bind metal ions, IMAC was used. The elution of AtLEA4-5 through the metal-bound columns with different metal ions was followed by SDS–PAGE. For Ca(II), Mn(II), and Fe(III) columns, the protein was found in the washed fraction, indicating that the protein did not bind these metal ions (Fig. S2). For Zn(II), Cu(II), and Ni(II) columns, the protein was eluted only when EDTA was added, showing that the protein is able to bind these metal ions (Fig. S2).

### Metal binding does not induce a change on secondary structure

**Figure 2:**
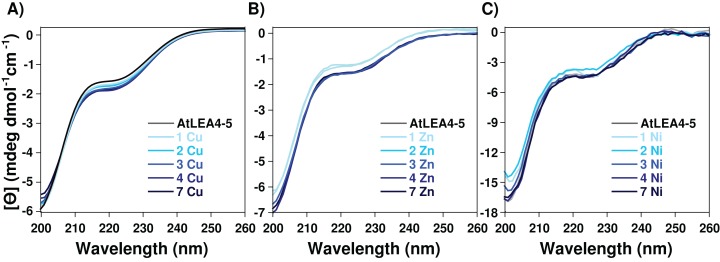
Effect of metal ions on AtLEA4-5 secondary structure. CD spectra of AtLEA4-5 titrated with (A) Cu(II), (B) Zn(II) and (C) Ni(II). Spectra of AtLEA4-5 in the absence (black line) or presence of 1, 2, 3, 4, and 7 equivalents of the metal ion (light to dark blue). The profiles did not undergo any significant variation after the addition of Cu(II), Zn(II) or Ni(II), suggesting that none of the metal ions induce changes in the AtLEA4-5 secondary structure under these conditions.

To determine whether the metal-ion binding to AtLEA4-5 protein induces secondary structure formation, AtLEA4-5 protein samples containing increasing amounts of Cu(II), Zn(II), and Ni(II) were analyzed by CD. CD analysis by the CAPITO (Wiedemann, Bellstedt & Görlach, 2013) revealed a pre-molten globule like-state (Figs. S1 and S3). This profile did not undergo any significant variation after the addition of Cu(II), Zn(II), and Ni(II), suggesting that none of the metal ions tested induces changes in AtLEA4-5 secondary structure under these conditions (Fig. 2 and Fig. S3).

### Histidine residues coordinate the binding to Cu(II)

**Figure 3:**
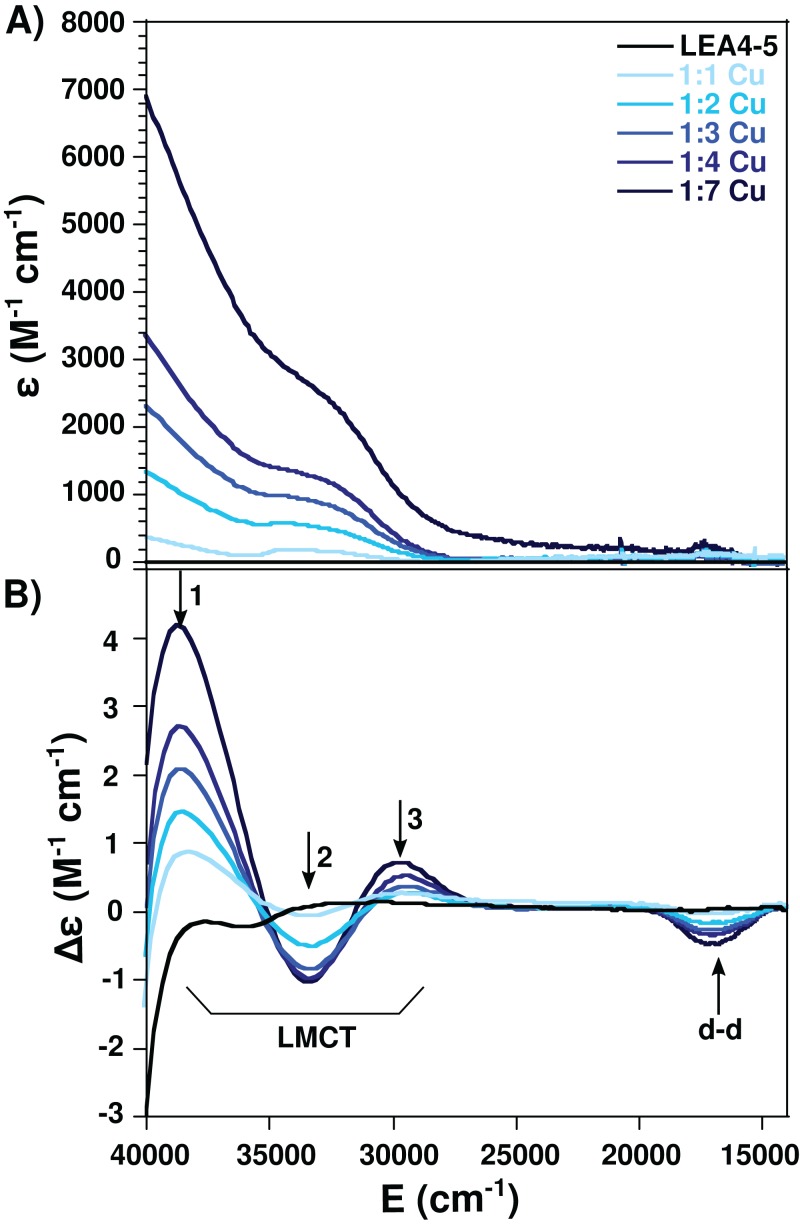
Cu(II)-AtLEA4-5 complex formation by electronic absorption. (A) UV–vis and (B) CD spectra of AtLEA4-5 in the absence (black line) or presence of 1, 2, 3, 4, and 7 equivalents of Cu(II) (light to dark blue). The arrows indicate the three ligand to metal charge transfer (LMCT) bands and the *d*–*d* bands. These bands correspond to transitions from the histidine imidazole group to copper and backbone deprotonated amides to copper.

Taking advantage of the paramagnetic properties of Cu(II), to further characterize the nature of the Cu(II) coordination to AtLEA4-5 we used electronic absorption (UV–vis and CD) in the UV–visible region. For this analysis AtLEA4-5 was titrated with different Cu(II) concentrations and the samples were analyzed. A negative band around 17,000 cm^−1^ (Δε = −1.98) corresponding to a *d–d* transition was observed by CD. Moreover, three ligand to metal charge transfer (LMCT) bands were detected, two positive: 29,300 cm^−1^ (Δε = 0.33) and 38,700 cm^−1^ (Δε = 1.05), and one negative: 33,500 cm^−1^, (Δε = −0.56) (Fig. 3). The positive bands correspond to transitions from histidine imidazole group to copper, π_1_ → Cu(II) (27,000–35,700 cm^−1^) and π_2_ → Cu(II) (32,500–40,800 cm^−1^), whereas the negative band corresponds to a transition from deprotonated amides to the metal, N → Cu(II) (31,000–34,000 cm^−1^)(Bryce, Roeske & Gurd, 1966; Bernarducci et al., 1981). These results indicate that AtLEA4-5 coordinates Cu(II) through at least one nitrogen of a histidine imidazole and backbone deprotonated amides.

**Figure 4:**
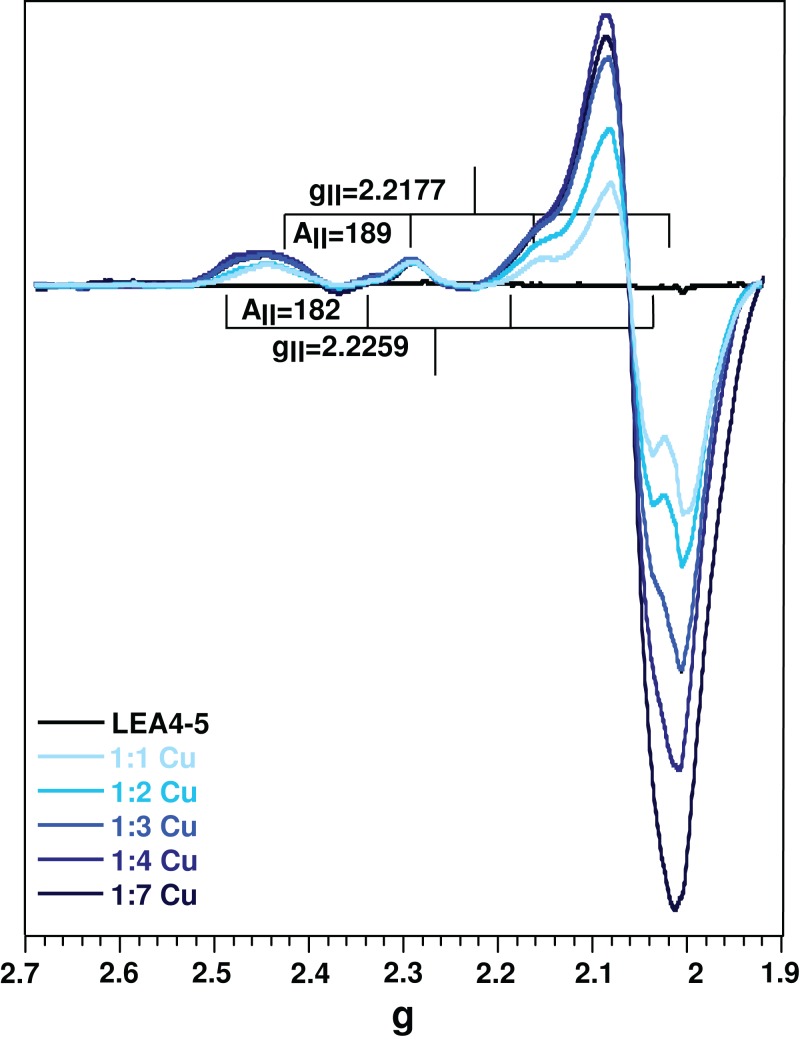
Cu(II)-AtLEA4-5 complex formation by EPR. EPR spectra of AtLEA4-5 in the absence (black line) or presence of 1, 2, 3, 4, and 7 equivalents of Cu(II) (light to dark blue). Two sets of signals were measured with *g*_ll_ = 2.2177 and *A*_ll_ = 188.5 and *g*_ll_ = 2.261 and *A*_ll_ = 182, corresponding to a 4N and N3O equatorial coordination, respectively. These results indicate that there are at least two species of the Cu(II)-AtLEA4-5 complex with two different equatorial coordination modes.

Electron paramagnetic resonance spectroscopy was used to detect molecular changes produced by the local environment of paramagnetic Cu(II). With the addition of one equivalent of Cu(II), the EPR spectra showed two sets of signals: *g*_ll_ = 2.2177/*A*_ll_ = 188.5, and *g*_ll_ = 2.261/*A*_ll_ = 182, corresponding to a 4N and N3O equatorial coordination mode, respectively, according to the Peisach and Blumberg plots (Peisach & Blumberg, 1974). The splitting of the signal in the perpendicular region (∼2.05) is also characteristic of a nitrogen coordination (Fig. 4) (Nunes et al., 2010). These results indicate that there are at least two species of the Cu(II)-AtLEA4-5 complex with two different equatorial coordination modes. The first with four nitrogen molecules, at least one from the imidazole of a histidine residue, while the others arise from the protein backbone deprotonated amides. The second mode involves one nitrogen, possibly from a histidine imidazole, and three oxygen molecules from protein carbonyl groups or from water molecules.

We estimated the binding parameters by plotting average EPR signals changes as function of Cu(II) concentrations. The data was fitted with to a hyperbolic equation and provided an apparent metal binding dissociation constant in the micromolar range (∼300 μM) (Fig. S4).

### Metal binding induces oligomerization

**Figure 5:**
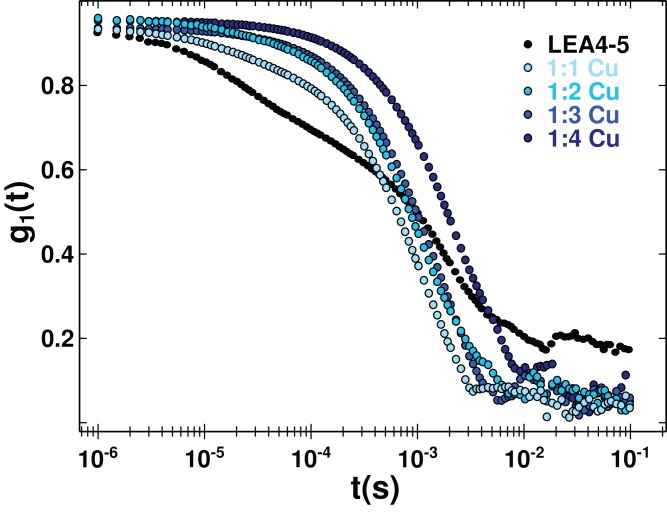
Protein oligomerization induced by metal binding. Correlation function of AtLEA4-5 in the absence (black line) or presence of 1, 2, 3, and 4 equivalents of Cu(II) (light to dark blue). The data were used to obtain translational diffusion coefficients by the cumulant and distribution methodologies (Fig. S5). Measurements of AtLEA4-5 in the presence of different Cu(II) concentrations yielded a shift to the right, indicating a size increase due to oligomerization induced by metal binding.

Even though, we did not find an effect of metal binding on AtLEA4-5 secondary structure, it was possible that this could affect its quaternary organization. In order to get insight of the oligomeric state of the metal complex, we perform DLS (Fig. 5 and Fig. S5). Although it is difficult to obtain an absolute size from an IDP, we can measure the diffusion coefficient and estimate the radius of a spherical molecule with that diffusion. As a reference, we used an empirical equation proposed to estimate the hydrodynamic radius from IDPs and calculated the corresponding translational diffusion coefficient (*R*_H_ = 3 nm, *D* = 8.2 × 10^−7^ cm^2^ s^−1^) for a monomer. The cellular concentration of LEA proteins is difficult to determine, due to the fact that they are differentially expressed during embryo-genesis and in response to water deficit (Olvera-Carrillo, Reyes & Covarrubias, 2011); nevertheless, a concentration of ∼250 μM has been reported (Roberts et al., 1993). The DLS measurements of AtLEA4-5 solution at 120 μM concentration show a polydisperse curve, from were could obtain three mayor components with apparent diffusion coefficient of *D* = 8.26 × 10^−6^, *D* = 1.95 × 10^−7^, and *D* = 0.07 × 10^−7^ cm^2^ s^−1^ which corresponds to apparent *R*_H_ of 2.98, 12.6, and 351 nm (Fig. S5). From this we could conclude that under these conditions AtLEA4-5 consist of different states, that include monomer, probably tetramers, and higher order oligomers. Lower protein concentration still result in polydisperse curves (Fig. S6). Different oligomeric states in solution have already been reported for some other LEA proteins (Rivera-Najera et al., 2014; Liu et al., 2017).

Although it is difficult to correlate the diffusion coefficient with the absolute size of the protein, it can be used reliably to reveal changes due to an oligomerization process. DLS measurements of AtLEA4-5 in the presence of different Cu(II) concentration yielded a decrease in the translational diffusion coefficient consistent with a size increase due to oligomerization induced by metal binding (Fig. 5). The diffusion coefficient obtained for the metal complexes described by one average population (cumulant method, Fig. S5) were: *D* = 0.134 × 10^−6^ cm^2^ s^−1^ 1:1 stoichiometry, *D* = 0.1 × 10^−6^ cm^2^ s^−1^ 1:2 stoichiometry, *D* = 0.06 × 10^−6^ cm^2^ s^−1^ 1:3 stoichiometry, and *D* = 0.03 × 10^−6^ cm^2^ s^−1^ 1:4 stoichiometry (Fig. S5). All of theses states would correspond to higher order oligomers.

### AtLEA4-5 presents moderate affinity for metal ions

**Figure 6:**
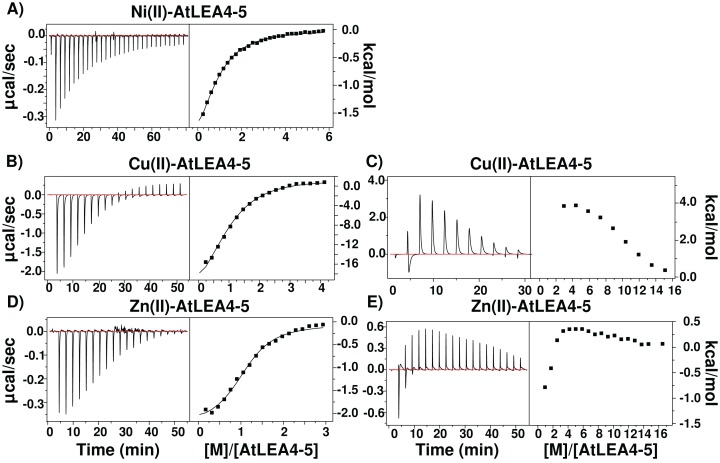
Metal binding by ITC. Isothermal titration calorimetry of AtLEA4-5 bound to (A) Ni(II), (B) Cu(II), and (C) Zn(II). The left side shows the experimental isothermic titrations; while the right side shows the reaction heat. In all cases the solid line represents the best fit to a binding model. (A) Ni(II) was best fitted to a single binding site model with a 1:1 stoichiometry. (B) and (C) Cu(II) and (D) and (E) Zn(II) exhibited a complex behavior involving both exothermic and endothermic processes and were fitted to multiple binding sites models. These transitions indicate the presence of at least two processes accounting for the different heat reactions.

Because IMAC procedure just yields qualitative information on proteins binding metal ions, we used ITC to obtain affinity values for the binding of AtLEA4-5 to Cu(II), Zn(II), and Ni(II). As shown in Fig. 6, the addition of Ni(II) produced a simple exothermic thermogram, which was best fitted to a single binding site model with a 1:1 stoichiometry; whereas the addition of Cu(II) and Zn(II) exhibited a complex behavior involving both exothermic and endothermic processes. At low equivalents of Cu(II) and Zn(II) the thermogram shows an exothermic heat reaction, which was followed by a late endothermic process as the metal concentration increases. This transition indicates the presence of at least two processes accounting for the different heat reactions (Fig. 6).

The resulting injection heats were fit with a binding model to estimate the binding affinity (*K*) and the enthalpy changes (Δ*H*). Whereas the free energy changes (Δ*G*) and the entropy changes (Δ*S*) were calculated by:

$$\Delta G = RT{\rm{ln}}K\;{\rm{ and }}\;\Delta G = \Delta H-T\Delta S$$

where *R* is the gas constant (1.987 cal Kmol^−1^) and *T* is the temperature in Kelvin (298 K).

In all cases, the negative enthalpies and the positive entropies for the first binding event (Table 1), indicated that the metal ion binding is enthalpic and entropic driven. In contrast, the positive Δ*H* and Δ*S* values for the endothermic reaction indicated a entropy-driven process. The largest apparent dissociation constants of AtLEA4-5 for each metal are in the micromolar range (Table 1) with a slightly higher affinity for Cu(II).

**Table 1:** Thermodynamic parameters obtained from ITC analysis to characterize the binding between AtLEA4-5 protein and Cu(II), Zn(II), and Ni(II).

Metal ion	*n* site	*K*_a_ (L/mol)	*K*_d_ (μ mol/L)	Δ*H* (kcal/mol)	Δ*S* (cal/mol/deg)	Δ*G* (kcal/mol)
Cu(II)	1	2 × 10^5^ ± 3 × 10^4^	6.4 ± 0.3	−20.7 ± 0.3	45.3	−7.2
	2	6 × 10^3^ ± 1 × 10^2^	179 ± 0.01	−9.2 ± 0.7	13.8	−5.1
	3	5 × 10^5^ ± 3 × 10^4^	1.8 ± 0.3	−32.9 ± 0.6	84.5	−7.7
Zn(II)	1	9 × 10^5^ ± 8 × 10^5^	3.3 ± 1.25	−2.1 ± 0.04	−20.1	−8.1
	2	3 × 10^4^ ± 4 × 10^3^	40 ± 25	−2.8 ± 0.4	−11.0	−6.1
Ni(II)	1	3 × 10^5^ ± 2 × 10^4^	9.1 ± 0.5	−13.9 ± 0.2	−23.5	−6.9

## Discussion

Ion sequestration by LEA proteins has been proposed as one of the possible functions in response to environmental stress such as cold, drought and high salinity (Krüger et al., 2002; Hara, Fujinaga & Kuboi, 2004; Liu et al., 2011, 2013). Most of the evidence comes from IMAC screening experiments and from antioxidant activity assays (Hara, Fujinaga & Kuboi, 2005; Liu et al., 2013; Hara et al., 2016). However, little is known about the characteristics of the interaction between these proteins and metal ions; such as the association affinity, the regions and residues involved, their thermodynamic properties and the effect on the protein structural organization.

In this work, we report that AtLEA4-5, an Arabidopsis group 4 LEA protein, is able to bind Cu(II), Zn(II), and Ni(II), but not Ca(II), Mn(II), or Fe(III) (Fig. S2). The binding affinities, determined by ITC, are in the micromolar range which is comparable to previously reported values for proteins involved in metal sequestering as metallothioneins or phytochelatins (Chekmeneva et al., 2008; Liu et al., 2011), suggesting that AtLEA4-5 protein may play a role as metal detoxification, and as modulator of cell ion homeostasis during water deficit. Our results also indicated that AtLEA4-5 protein contains a single binding site for Ni(II), while Zn(II) and Cu(II) have multiple binding sites and promote oligomerization.

The data reported here showed that metal-protein complex formation does not induce a change in the protein secondary structure (α-helix or β-sheet) (Fig. 2; Fig. S3), and hence the protein probably is just forming loops mediated by the metal or multiple protein molecules are binding the same metal. In contrast to the classical binding paradigm for globular protein, the lack of a stable secondary structure formation and the moderate affinity, suggests that an ensemble of conformations remains accessible to the metal for binding, favoring different types of metal association to specific protein sites. The formation of multiple binding modes with moderate affinity has been reported for other metal binding IDPs (Chekmeneva et al., 2008; Liu et al., 2011; Sacco et al., 2012).

It has been suggested that histidine residues could be the putative anchoring site for metal binding (Gusman et al., 2001). On this regard, AtLEA4-5 has a high content of histidines (1 in the *N*-terminal domain and 6 in the C-terminal domain). Moreover, electronic absorption (UV–vis and CD) data showed LMCTs characteristics of imidizole groups. Whereas EPR results indicate that there are at least, two coordination modes between the protein and Cu(II). The first one includes four nitrogen atoms which bind equatorially to Cu(II), at least one from a histidine imidazole, and the others from backbone deprotonated amides. While the second coordination mode includes one nitrogen, possibly from a histidine’s imidazole, and three oxygens from backbone carbonyl group or water molecules (Bernarducci et al., 1981). The presence of a broad band from 540 to 650 nm in the CD spectra is also consistent with a mixture of species with different coordination modes (Nunes et al., 2010).

Self-assembly induced by metal ion binding has been reported for other IDPs such as alpha-synuclein (Uversky, Li & Fink, 2001), prion (Bocharova et al., 2005), Tau (Mo et al., 2009), and Aβ(1–42) peptide (Bonda et al., 2011; Breydo & Uversky, 2011). As indicated by DLS data, a similar behavior was observed for AtLEA4-5, with a decrease in the diffusion coefficient as Cu(II) concentration increased, implying the formation of oligomers mediated by metal binding. Recently, it has been shown for GmPM1, a LEA4 group member from soybean, that metal binding induces oligomerization probably involving His residues as detected by chemical cross-linking (Liu et al., 2017). Even though the role in planta of this metal binding effect on LEA protein structural organization is unknown, this may be related to a different mechanism to enforce their function as protectors of macromolecules or cellular structures, and/or as water organizers under extreme low water availability, as it occurs in dry seeds or in dehydrated resurrection plants, conditions where consequently metal concentrations tend to increase (Otegui, 2002; Kranner & Colville, 2011). This effect may also constitute a mechanism for LEA proteins to act as metal detoxifiers.

Interestingly, GmPM1 proteins bind Fe(III) (Liu et al., 2011), whereas we could not detect binding of AtLEA4-5 to this metal ion. The sequence similarity between AtLEA4-5 and GmPM1 is about 58% containing both of them several His residues (Fig. S7). And while both proteins are able to bind Cu(II), they differ in the binding capabilities for Fe(III). This suggests that, even though, many LEA proteins could be able to bind metal ions and form oligomers, there is not necessarily a conserved specificity for metal ion binding among LEA proteins, even from the same group. The different ion binding properties have to be further study to understand the relation to plant survival.

To gain insight into the thermodynamic of metal ion binding, we performed ITC experiments. This approach allows to directly measure the enthalpy changes, and obtain the association constants by a least-squares fit to a binding model. Then we calculate the free energy and the entropy changes. Ni(II) binding to AtLEA4-5 presents a single exothermic transition with a stoichiometric equivalence of 1:1, suggesting one binding site. Whereas, the thermograms obtained by the titration of Zn(II) and Cu(II) showed a complex process with an initial exothermic event followed by a late endothermic reaction (Fig. 6). The analysis of this type of curves is complicated due to the fact that it reflects contributions from different process.

Taking into consideration the spectroscopic data, that showed that AtLEA4-5 is able to bind several equivalents of metal in at least two different configurations, and that the metal binding induces the formation of oligomeric species, the thermogram must arise from a complicated interplay between the coordination chemistry of metal binding and the induced self-association. Based on this qualitative assessment, the binding isotherm was fitted to a multiple process model.

Even-though, the complexity of the process prevents us from completely dissecting the different contributions of the measured data, we can assume that the first exothermic event is describing the metal binding while the following events correspond to a mixture of subsequent metal ion binding and oligomerization. The observed favorable enthalpy in the first event, primarily reflects protein metal ion interactions, whereas the change in the binding entropy is related to metal ion desolvation. The following process is entropically controlled, which is likely due to the rearrangement of water surrounding the proteins and disruption of the metal ion hydration sphere upon metal binding and oligomerization.

While the ITC measures the enthalpy changes for any process (binding and oligomerization), the spectroscopic data are only sensitive to the metal binding and not to the oligomerization. Therefore, we used the EPR signals from the metal titration to estimate an apparent dissociation constant. The binding information obtained by the spectroscopy values is consistent with the ITC data with an apparent Kd binding in the μM range (Fig. S4). Under normal conditions, the metal ions concentration in plants has been reported in a low micromolar range (Álvarez-Fernández et al., 2014), suggesting a plausible physiologically relevant interaction between AtLEA4-5 and metal ions.

Previous reports have suggested that plants with silenced LEA 4 genes increase their susceptibility to oxidative stress (Senthil-Kumar & Udayakumar, 2006). This evidence, together with our results, particularly for Cu(II), suggests that AtLEA4-5 protein could have a protective role under oxidative stress. The Cu-AtLEA4-5 complex can act as a redox center, and consequently it may be responsible for antioxidant activity, scavenging metal ions or ROS under various stress conditions (Liu et al., 2013; Hara, Kondo & Kato, 2013). Due to the fact that all these proteins accumulate under water deficit conditions, these observations also could indicate that one of the deleterious effects of this adverse environment is the accumulation of metal ions, and that LEA proteins not only prevent the impairment of functional protein structures but also are able to counteract the detrimental outcome of metal ion increasing concentrations in cells. Metal ions accumulation could causes damage to metabolism, genetic expressions and structure of proteins. Also, physiological changes such as size and mass reductions has been reported when plants are treated with metals in the micro–millimolar range (Kranner & Colville, 2011).

Taking into account that the *N*-terminal region of AtLEA4-5 was able to fold and prevent enzyme unfolding, and that most of the His residues are located in the *C*-terminal region, it seems reasonable to think that while the amino terminal region could be responsible for protecting enzymatic activity by binding and adopting secondary structure features, the carboxy terminal region participates in metal sequestration reducing the generation of ROS.

## Conclusion

**Figure 7:**
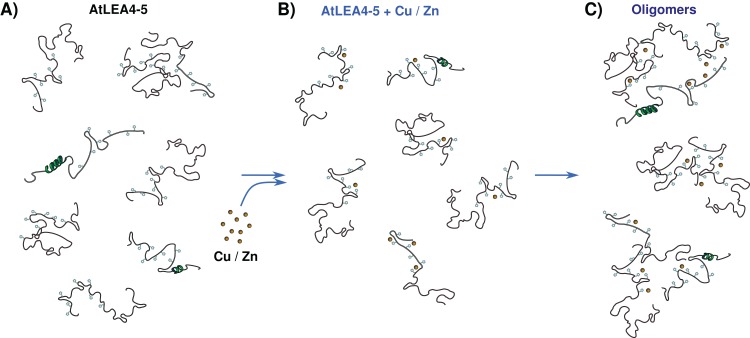
Metal binding model. (A) An ensemble of conformations is accessible for binding. (B) Metals interact with moderate affinity mainly with His residues, with multiple forms and multiple binding sites, without forming a unique well-defined binding site. (C) As metal ion concentration increases their binding to the protein induces the formation of oligomeric species.

The data presented here allow us to propose a metal binding model, where the ensemble of conformations remains accessible upon binding (Fig. 7). The metals interact mainly with His residues with moderate affinity in different conformations and multiple binding sites. These different coordination modes, without forming a unique well-defined binding site, may indicate a fuzzy complex. A clouds-interaction model between IDPs and small-molecule ligands has been proposed (Jin et al., 2013); however, in this case it seems that the metal ions binds preferentially to His residues which, upon an increase in metal ion concentration, induces the formation of higher oligomeric species.

Our results support the multifunctionality of LEA proteins and suggest that the AtLEA4-5 protein might perform both protection roles, preventing loss of protein activity and metal scavenger under low water conditions shedding light on the functions of group 4 LEA proteins. The exact number and sequence of each binding site and the corresponding affinities is still to be determined.

## Supplemental Information

10.7717/peerj.4930/supp-1 Supplemental Information 1 Fig. S1. Evaluation of the intrinsic disorder propensities of AtLEA4-5. (A) AtLEA4-5 sequence. Positive charged residues are in blue, negative charged residues are in red, glycines are in green and histidine residues are in purple. The N-terminal domain and the C-terminal domain are shown. (B) Predicted disorder probability of AtLEA4-5 by IUPred and PONDR methods. Both methodologies show disorder probability values above the disorder threshold (score = 0.5). (C) Classification of AtLEA4-5 by Intrinsically Disordered Ensemble Relationships (CIDER) methods. R_1_ region correspond to weak polyampholytes and polyelectrolytes (globules and tadpoles), R_2_ region correspond to Janus sequences (collapsed or expanded-context dependent), R_3_ region correspond to strong polyampholytes (coils, hairpins and chimeras), R_4_ region correspond to negatively charged strong polyelectrolytes (coils, hairpins and chimeras) and R_5_ region correspond to positively charged strong polyelectrolytes (swollen coils). AtLEA4-5 is situated in region R_1_. (D) CD spectra of AtLEA4-5, showing a typical CD spectrum for a disorder protein, as indicated by the presence of minimum at ~200 nm. Secondary structure deconvolution of AtLEA4-5 is shown. (E) Analysis of CD data using CAPITO. AtLEA4-5 data is located in region close to a pre molten globule structure classification. Accordingly, AtLEA4-5 contains some secondary structure elements but does not has a stable folded structure.
.- Zsuzsanna Dosztányi, Veronika Csizmók, Péter Tompa and István Simon, 2005, IUPred: web server for the prediction of intrinsically unstructured regions of proteins based on estimated energy content. *Bioinformatics* 21, 3433–3434.
.- Xue B, Dunbrack RL, Williams RW, Dunker AK, Uversky VN., 2010, PONDR-FIT: a meta-predictor of intrinsically disordered amino acids. *Biochem. Biophys. Acta.* 1804:996–1010.
.- Holehouse, A.S., Ahad, J., Das, R.K., and Pappu, R.V., 2015, CIDER: Classification of Intrinsically Disordered Ensemble Regions. *Biophys. J.* 108, 228a.
.-Wiedemann C, Bellstedt P, Görlach M (2013). CAPITO–A web server based analysis and plotting tool for circular dichroism data. Bioinformatics 29(14): 1750–1757

10.7717/peerj.4930/supp-2 Supplemental Information 2 Fig. S2. AtLEA4-5 interaction with metal ions by qualitative method IMAC. SDS-PAGE of AtLEA4-5 eluted through columns with (A) Ca(II), (B) Fe(III), (C) Mn(II), (D) Cu(II), (E) Zn(II) and (F) Ni (II). Lines 2–5 correspond to washed fractions. Lines 6–9 correspond to EDTA 50 mM eluted fractions. (G) Shows IMAC controls with BSA that is reported to bind Cu(II) but not Ca(II) and Mn(II).

10.7717/peerj.4930/supp-3 Supplemental Information 3 Fig. S3. Effect of metal ions on AtLEA4-5 secondary structure. Analysis of CD data of AtLEA4-5 in the absence (black) and presence of 7 molar equivalents (light blue) of (A) Cu(II), (B) Zn(II) and (C) Ni(II) using CAPITO method. AtLEA4-5 in presence of three metal keeps its pre molten globule structure.
-.Wiedemann C, Bellstedt P, Görlach M., 2013, CAPITO–A web server based analysis and plotting tool for circular dichroism data. *Bioinformatics*, 29(14): 1750–1757

10.7717/peerj.4930/supp-4 Supplemental Information 4 Fig. S4. Estimation of Kd from EPR data. (A) EPR spectra of AtLEA4-5 in the absence (black line) or presence of 0.5, 1, 1.5, 2, 2.5, 3, 3.5, 4, 5 and 7 equivalents of Cu(II) (light to dark blue). The changes of intensities at *g* = 2.45 and *g* = 2.15 were selected to estimating the binding constant. (B) Normalized signal from the average of *g* = 2.45 and *g* = 2.15 plotted as a function of Cu(II) equivalents. The data was fitted to a hyperbolic equation (red line), where y is the absorbance, [Cu] is the copper concentration, p is the maximum specific binding, and k_b_ is the apparent binding constant.

10.7717/peerj.4930/supp-5 Supplemental Information 5 Fig. S5. Dynamic light scattering (DLS) analysis. DLS was used to obtain changes in the oligomer state through measurement of the translational diffusion coefficients. An estimation of the hydrodynamic radius (R_H_) from a spheric molecule with the same diffusion can be obtained using the Stokes-Einstein equation. The diffusion coefficient was obtained from the correlation function. The cumulant method assumes a single population of particles with an exponential decay. The first “cumulant” corresponds to an average diffusion coefficient while the second correspond to the variance. In a sample with molecules of different sizes or shapes (polydisperse), to describe the correlation function it is necessary multiple exponential decays fitted with a non-linear methodology. The distribution method generates a distribution of molecules with different hydrodynamic properties which can be described by individual parameters. DLS signal is very sensitive to the presence of large molecules due to the fact that the scattered light is proportional to the diameter to the power of six . (A) DLS measurements of AtLEA4-5 solutions shown a polidisperse curve which was not well describe by a single population (cumulant method). The best fitting was obtained by a distribution with three different populations. While AtLEA4-5 in the presence of (B) 1, (C) 2, (D) 3, and (E) 4 equivalents of Cu(II), were better described by the cumulant method as seen by the quality of the fitting. Although the data does not allow to determine precisely the sizes of the oligomers, it reveal a consistent multimerization of AtLEA4-5 by the addition of Cu(II). Residuals and the fitted parameters are shown.

10.7717/peerj.4930/supp-6 Supplemental Information 6 Fig. S6. Protein concentration effect on the oligomeric populations. (A) DLS measurements of AtLEA4-5 solutions at 50 μM and (B) at 12μM. Lower protein concentration still result in polidisperse curves, with the best fitting obtained by a distribution of several different populations. This suggest that the protein has different oligomeric states in solution.

10.7717/peerj.4930/supp-7 Supplemental Information 7 Fig. S7. Sequence alignment for AtLEA4-5, GmPM1 and GmPM9. Fully conserved residues are contained in black boxes, positive charged residues are in blue, negative charged residues are in red, glycines are in green and histidine residues are in purple.

## Additional Information and Declarations

### Data Availability

The following information was supplied regarding data availability: The raw data are provided in the Supplemental Files.
