# Supplementary figures and images for: Metal-binding polymorphism in late embryogenesis abundant protein AtLEA4-5, an intrinsically disordered protein
Source: PeerJ. 2018 Jun 7;6:e4930. doi: 10.7717/peerj.4930 (PMC5994335; doi:10.7717/peerj.4930)

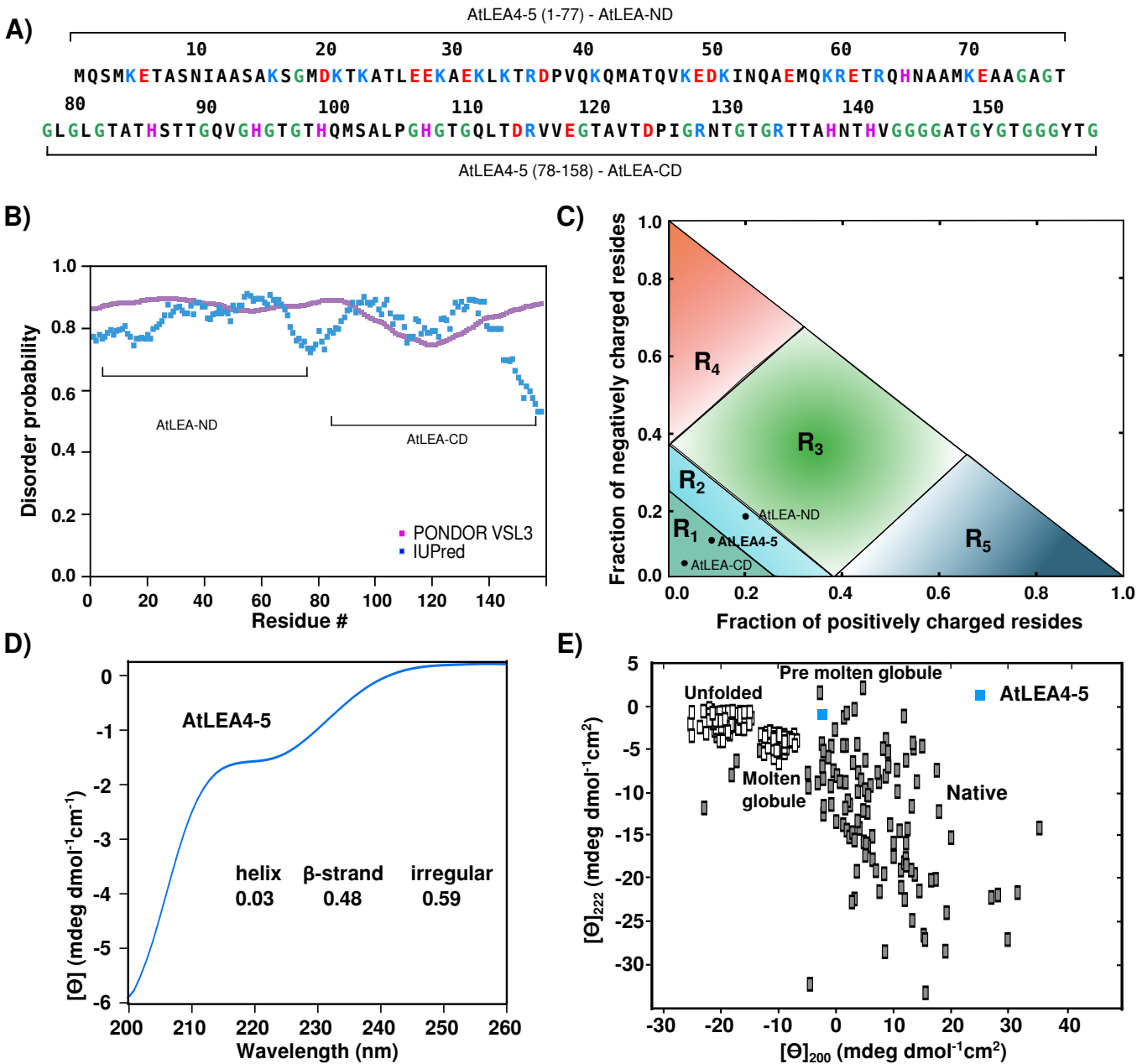

Supplement: Supplemental Information 1 — (A) AtLEA4-5 sequence. Positive charged residues are in blue, negative charged residues are in red, glycines are in green and histidine residues are in purple. The N-terminal domain and the C-terminal domain are shown. (B) Predicted disorder probability of AtLEA4-5 by IUPred and PONDR methods. Both methodologies show disorder probability values above the disorder threshold (score = 0.5). (C) Classification of AtLEA4-5 by Intrinsically Disordered Ensemble Relationships (CIDER) methods. R1 region correspond to weak polyampholytes and polyelectrolytes (globules and tadpoles), R2 region correspond to Janus sequences (collapsed or expanded-context dependent), R3 region correspond to strong polyampholytes (coils, hairpins and chimeras), R4 region correspond to negatively charged strong polyelectrolytes (coils, hairpins and chimeras) and R5 region correspond to positively charged strong polyelectrolytes (swollen coils). AtLEA4-5 is situated in region R1. (D) CD spectra of AtLEA4-5, showing a typical CD spectrum for a disorder protein, as indicated by the presence of minimum at ~200 nm. Secondary structure deconvolution of AtLEA4-5 is shown. (E) Analysis of CD data using CAPITO. AtLEA4-5 data is located in region close to a pre molten globule structure classification. Accordingly, AtLEA4-5 contains some secondary structure elements but does not has a stable folded structure. .- Zsuzsanna Dosztányi, Veronika Csizmók, Péter Tompa and István Simon, 2005, IUPred: web server for the prediction of intrinsically unstructured regions of proteins based on estimated energy content. Bioinformatics 21, 3433–3434. .- Xue B, Dunbrack RL, Williams RW, Dunker AK, Uversky VN., 2010, PONDR-FIT: a meta-predictor of intrinsically disordered amino acids. Biochem. Biophys. Acta. 1804:996–1010. .- Holehouse, A.S., Ahad, J., Das, R.K., and Pappu, R.V., 2015, CIDER: Classification of Intrinsically Disordered Ensemble Regions. Biophys. J. 108, 228a. .-Wiedemann C, Bellstedt P, Görlach M (2013). CAPI [file peerj-06-4930-s001.pdf]

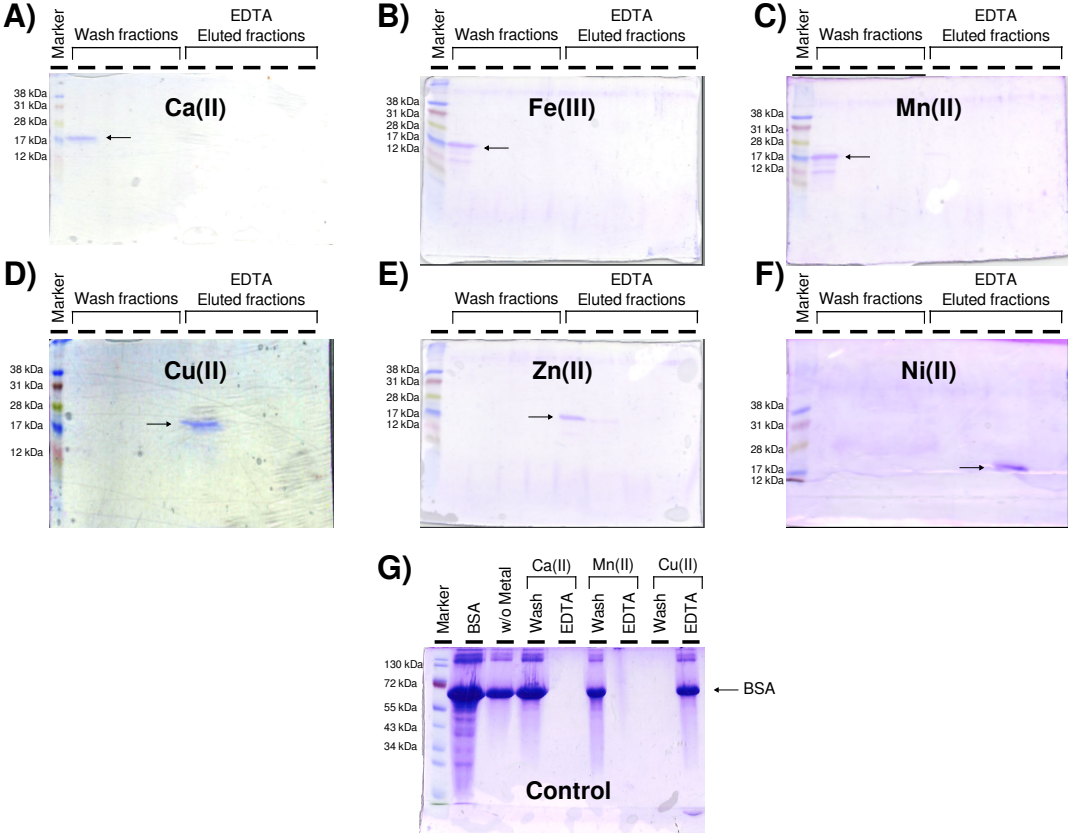

Supplement: Supplemental Information 2 — SDS-PAGE of AtLEA4-5 eluted through columns with (A) Ca(II), (B) Fe(III), (C) Mn(II), (D) Cu(II), (E) Zn(II) and (F) Ni (II). Lines 2–5 correspond to washed fractions. Lines 6–9 correspond to EDTA 50 mM eluted fractions. (G) Shows IMAC controls with BSA that is reported to bind Cu(II) but not Ca(II) and Mn(II). [file peerj-06-4930-s002.pdf]

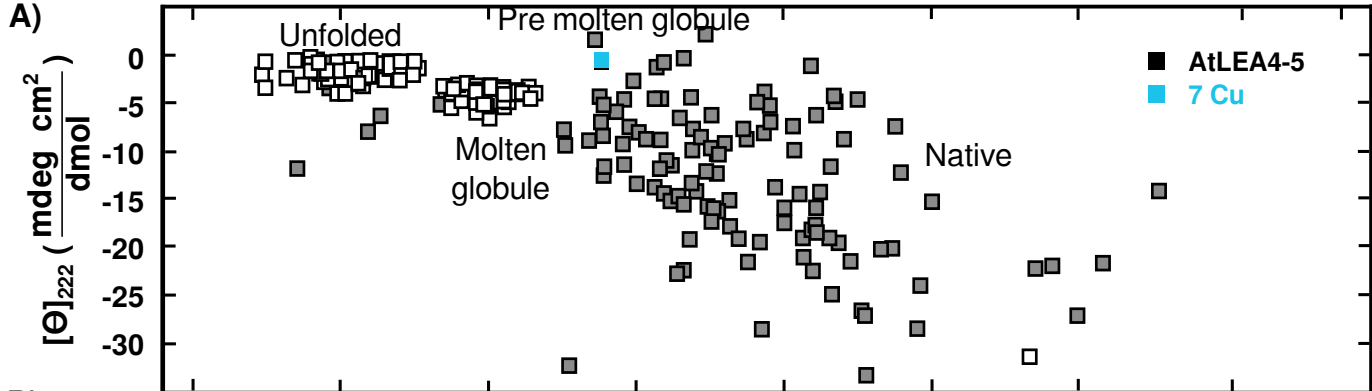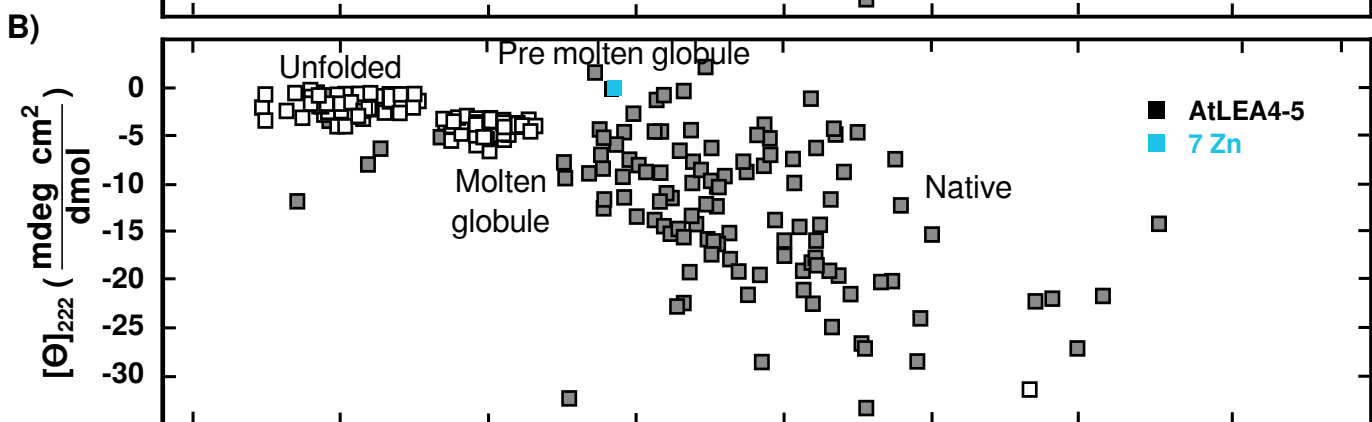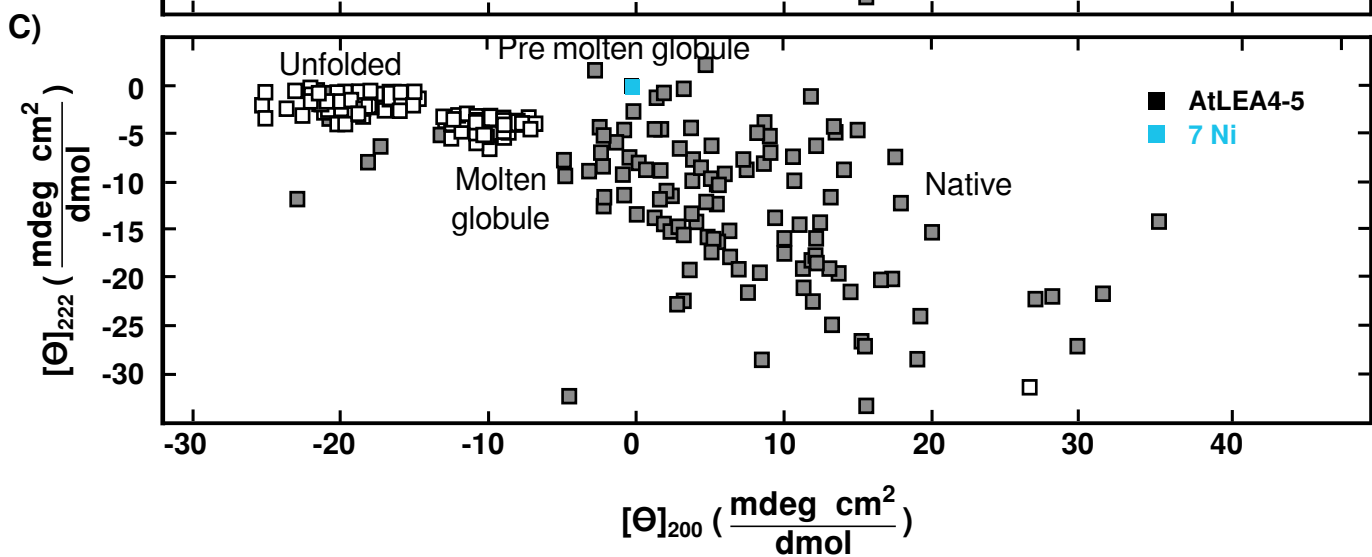

Supplement: Supplemental Information 3 — Analysis of CD data of AtLEA4-5 in the absence (black) and presence of 7 molar equivalents (light blue) of (A) Cu(II), (B) Zn(II) and (C) Ni(II) using CAPITO method. AtLEA4-5 in presence of three metal keeps its pre molten globule structure. -.Wiedemann C, Bellstedt P, Görlach M., 2013, CAPITO–A web server based analysis and plotting tool for circular dichroism data. Bioinformatics, 29(14): 1750–1757 [file peerj-06-4930-s003.pdf]

A)

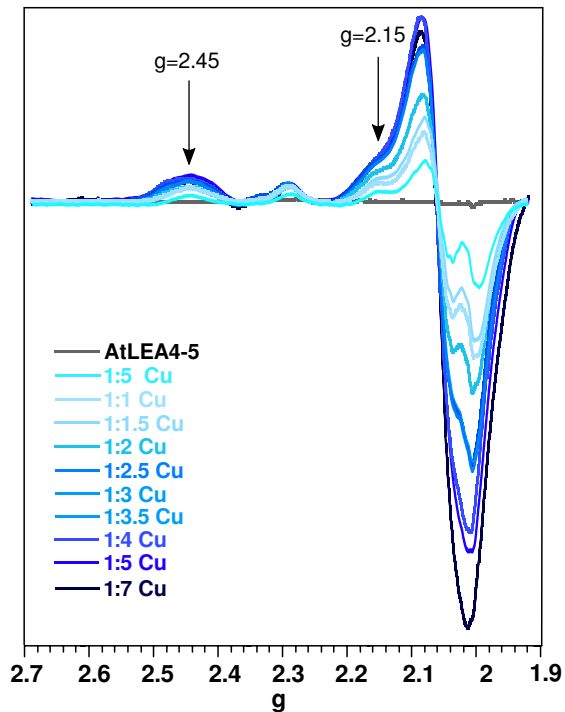

B)

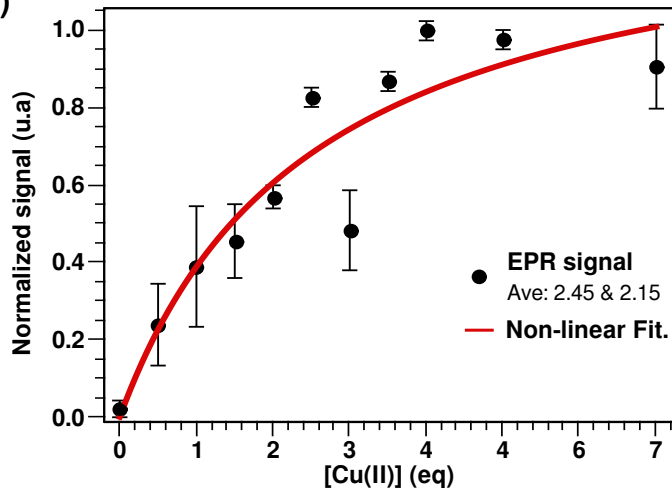

Non-linear Fit.

$$y = \frac{p[\text{Cu}]}{k + [\text{Cu}]}$$

$$k = 307 \pm 130 \mu\text{M}$$

$$p = 1.3 \pm 0.2$$

$$\text{Chi}^2/\text{doF} = 0.015$$

$$R^2 = 0.869$$

Supplement: Supplemental Information 4 — (A) EPR spectra of AtLEA4-5 in the absence (black line) or presence of 0.5, 1, 1.5, 2, 2.5, 3, 3.5, 4, 5 and 7 equivalents of Cu(II) (light to dark blue). The changes of intensities at g = 2.45 and g = 2.15 were selected to estimating the binding constant. (B) Normalized signal from the average of g = 2.45 and g = 2.15 plotted as a function of Cu(II) equivalents. The data was fitted to a hyperbolic equation (red line), where y is the absorbance, [Cu] is the copper concentration, p is the maximum specific binding, and kb is the apparent binding constant. [file peerj-06-4930-s004.pdf]
