# Supplementary material for: Metal-binding polymorphism in late embryogenesis abundant protein AtLEA4-5, an intrinsically disordered protein
Source: PeerJ. 2018 Jun 7;6:e4930. doi: 10.7717/peerj.4930 (PMC5994335; doi:10.7717/peerj.4930)
Supplement: Supplemental Information 5 — DLS was used to obtain changes in the oligomer state through measurement of the translational diffusion coefficients. An estimation of the hydrodynamic radius (RH) from a spheric molecule with the same diffusion can be obtained using the Stokes-Einstein equation. The diffusion coefficient was obtained from the correlation function. The cumulant method assumes a single population of particles with an exponential decay. The first “cumulant” corresponds to an average diffusion coefficient while the second correspond to the variance. In a sample with molecules of different sizes or shapes (polydisperse), to describe the correlation function it is necessary multiple exponential decays fitted with a non-linear methodology. The distribution method generates a distribution of molecules with different hydrodynamic properties which can be described by individual parameters. DLS signal is very sensitive to the presence of large molecules due to the fact that the scattered light is proportional to the diameter to the power of six . (A) DLS measurements of AtLEA4-5 solutions shown a polidisperse curve which was not well describe by a single population (cumulant method). The best fitting was obtained by a distribution with three different populations. While AtLEA4-5 in the presence of (B) 1, (C) 2, (D) 3, and (E) 4 equivalents of Cu(II), were better described by the cumulant method as seen by the quality of the fitting. Although the data does not allow to determine precisely the sizes of the oligomers, it reveal a consistent multimerization of AtLEA4-5 by the addition of Cu(II). Residuals and the fitted parameters are shown. [file peerj-06-4930-s005.pdf]

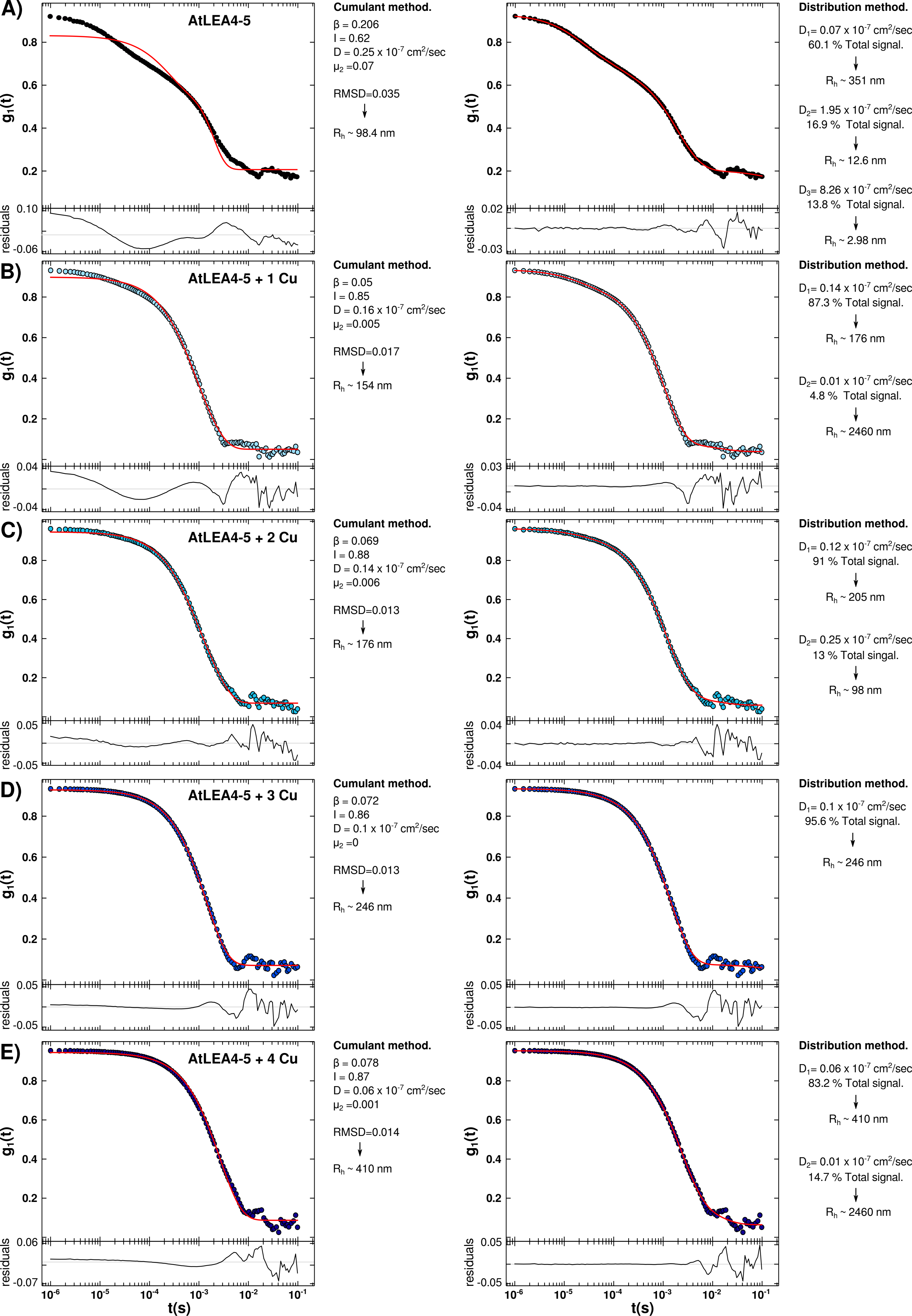

A)
AtLEA4-5
Cumulant method.
β = 0.206
I = 0.62
D = 0.25 x 10-7 cm2/sec
μ2 =0.07
RMSD=0.035
Rh ~ 98.4 nm
Distribution method.
D1= 0.07 x 10-7 cm2/sec
60.1 % Total signal.
Rh ~ 351 nm
D2= 1.95 x 10-7 cm2/sec
16.9 % Total signal.
Rh ~ 12.6 nm
D3= 8.26 x 10-7 cm2/sec
13.8 % Total signal.
Rh ~ 2.98 nm
B)
AtLEA4-5 + 1 Cu
Cumulant method.
β = 0.05
I = 0.85
D = 0.16 x 10-7 cm2/sec
μ2 =0.005
RMSD=0.017
Rh ~ 154 nm
Distribution method.
D1= 0.14 x 10-7 cm2/sec
87.3 % Total signal.
Rh ~ 176 nm
D2= 0.01 x 10-7 cm2/sec
4.8 % Total signal.
Rh ~ 2460 nm
C)
AtLEA4-5 + 2 Cu
Cumulant method.
β = 0.069
I = 0.88
D = 0.14 x 10-7 cm2/sec
μ2 =0.006
RMSD=0.013
Rh ~ 176 nm
Distribution method.
D1= 0.12 x 10-7 cm2/sec
91 % Total signal.
Rh ~ 205 nm
D2= 0.25 x 10-7 cm2/sec
13 % Total singal.
Rh ~ 98 nm
D)
AtLEA4-5 + 3 Cu
Cumulant method.
β = 0.072
I = 0.86
D = 0.1 x 10-7 cm2/sec
μ2 =0
RMSD=0.013
Rh ~ 246 nm
Distribution method.
D1= 0.1 x 10-7 cm2/sec
95.6 % Total signal.
Rh ~ 246 nm
E)
AtLEA4-5 + 4 Cu
Cumulant method.
β = 0.078
I = 0.87
D = 0.06 x 10-7 cm2/sec
μ2 =0.001
RMSD=0.014
Rh ~ 410 nm
Distribution method.
D1= 0.06 x 10-7 cm2/sec
83.2 % Total signal.
Rh ~ 410 nm
D2= 0.01 x 10-7 cm2/sec
14.7 % Total signal.
Rh ~ 2460 nm
g1(t)
residuals
t(s)
10-6
10-5
10-4
10-3
10-2
10-1
