# Supplementary material for: Metal-binding polymorphism in late embryogenesis abundant protein AtLEA4-5, an intrinsically disordered protein
Source: PeerJ. 2018 Jun 7;6:e4930. doi: 10.7717/peerj.4930 (PMC5994335; doi:10.7717/peerj.4930)
Supplement: Supplemental Information 6 — (A) DLS measurements of AtLEA4-5 solutions at 50 μM and (B) at 12μM. Lower protein concentration still result in polidisperse curves, with the best fitting obtained by a distribution of several different populations. This suggest that the protein has different oligomeric states in solution. [file peerj-06-4930-s006.pdf]

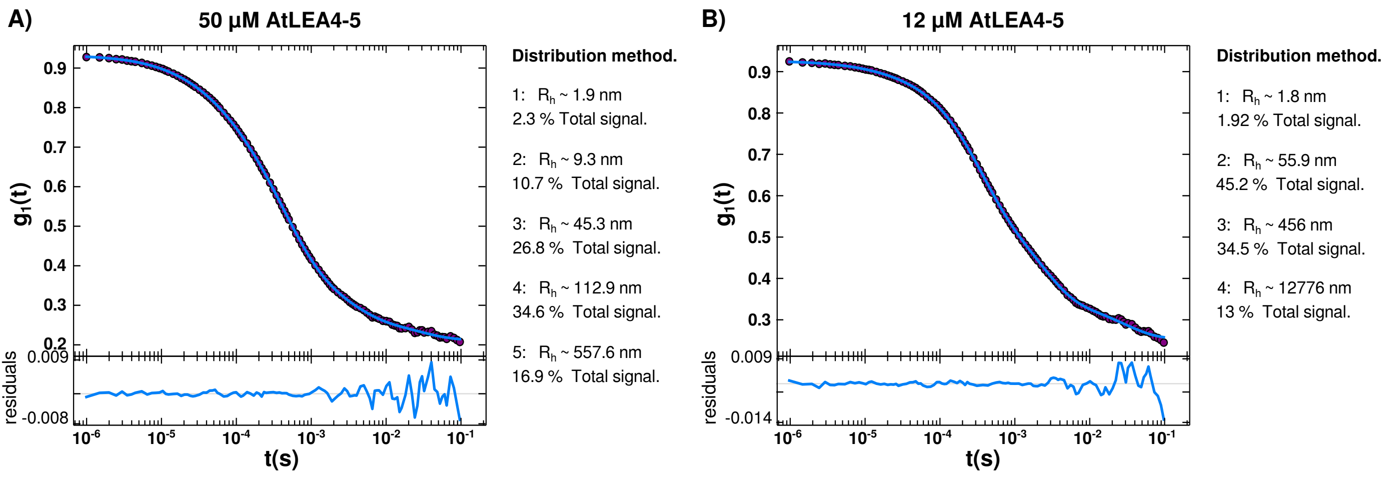
A)
50 µM AtLEA4-5
g1(t)
0.9
0.8
0.7
0.6
0.5
0.4
0.3
0.2
residuals
0.009
-0.008
10-6
10-5
10-4
10-3
10-2
10-1
t(s)
Distribution method.
1: Rh ~ 1.9 nm
2.3 % Total signal.
2: Rh ~ 9.3 nm
10.7 % Total signal.
3: Rh ~ 45.3 nm
26.8 % Total signal.
4: Rh ~ 112.9 nm
34.6 % Total signal.
5: Rh ~ 557.6 nm
16.9 % Total signal.
B)
12 µM AtLEA4-5
g1(t)
0.9
0.8
0.7
0.6
0.5
0.4
0.3
residuals
0.009
-0.014
10-6
10-5
10-4
10-3
10-2
10-1
t(s)
Distribution method.
1: Rh ~ 1.8 nm
1.92 % Total signal.
2: Rh ~ 55.9 nm
45.2 % Total signal.
3: Rh ~ 456 nm
34.5 % Total signal.
4: Rh ~ 12776 nm
13 % Total signal.
