# Supplementary material for: Metal-binding polymorphism in late embryogenesis abundant protein AtLEA4-5, an intrinsically disordered protein
Source: PeerJ. 2018 Jun 7;6:e4930. doi: 10.7717/peerj.4930 (PMC5994335; doi:10.7717/peerj.4930)
Supplement: Supplemental Information 7 — Fully conserved residues are contained in black boxes, positive charged residues are in blue, negative charged residues are in red, glycines are in green and histidine residues are in purple. [file peerj-06-4930-s007.pdf]

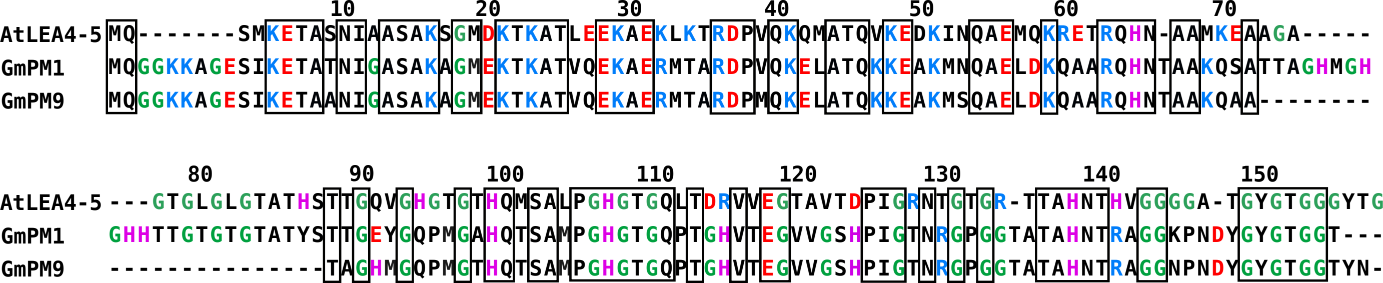
10 20 30 40 50 60 70
AtLEA4-5 MQ-------SMKETASNIAASAKSGMDKTKATLEEKAEKLKTRDPVQKQMATQVKEDKINQAEMQKRETRQHN-AAMKEAAGA-----
GmPM1 MQGGKKAGESIKETATNIGASAKAGMEKTKATVQEKAERMTARDPVQKELATQKKEAKMNQAELDKQAARQHNTAAKQSATTAGHMGH
GmPM9 MQGGKKAGESIKETAANIGASAKAGMEKTKATVQEKAERMTARDPMQKELATQKKEAKMSQAELDKQAARQHNTAAKQAA--------
80 90 100 110 120 130 140 150
AtLEA4-5 ---GTGLGLGTATHSTTGQVGHGTGTHQMSALPGHGTGQLTDRVVEGTAVTDPIGRNTGTGR-TTAHNTHVGGGGA-TGYGTGGGYTG
GmPM1 GHHTTGTGTGTATYSTTGEYGQPMGAHQTSAMPGHGTGQPTGHVTEGVVGSHPIGTNRGPGGTATAHNTRAGGKPNDYGYGTGGT---
GmPM9 ---------------TAGHMGQPMGTHQTSAMPGHGTGQPTGHVTEGVVGSHPIGTNRGPGGTATAHNTRAGGNPNDYGYGTGGTYN-
